# Work-Family Life Courses and Metabolic Markers in the MRC National Survey of Health and Development

**DOI:** 10.1371/journal.pone.0161923

**Published:** 2016-08-26

**Authors:** Rebecca E. Lacey, Meena Kumari, Amanda Sacker, Mai Stafford, Diana Kuh, Anne McMunn

**Affiliations:** 1 Department of Epidemiology and Public Health, University College London, London, United Kingdom; 2 Institute for Social and Economic Research, University of Essex, Colchester, United Kingdom; 3 Medical Research Council Unit for Lifelong Health and Ageing, University College London, London, United Kingdom; Medical University Innsbruck, AUSTRIA

## Abstract

The aim was to investigate whether the combined work-family life courses of British men and women were associated with differences in metabolic markers—waist circumference, blood pressure, high density lipoprotein cholesterol, triglycerides, and glycated haemoglobin—in mid-life. We used data from the Medical Research Council’s National Survey of Health and Development—the 1946 British birth cohort. Multi-channel sequence analysis was used to create a typology of eight work-family life course types combining information on work, partnerships and parenthood between ages 16–51. Linear regression tested associations between work-family types and metabolic outcomes at age 53 on multiply imputed data (20 imputations) of >2,400 participants. Compared with men with strong ties to employment and early transitions to family life, men who made later transitions to parenthood and maintained strong ties to paid work had smaller waist circumferences (-2.16cm, 95% CI: -3.73, -0.59), lower triglycerides (9.78% lower, 95% CI: 0.81, 17.94) and lower blood pressure (systolic: -4.03mmHg, 95% CI: -6.93, -1.13; diastolic: -2.34mmHg, 95% CI: -4.15, -0.53). Married men and women who didn’t have children had increased high density lipoprotein cholesterol (7.23% higher, 95% CI: 0.68, 14.21) and lower waist circumferences (-4.67cm, 95% CI: -8.37, -0.97), respectively. For men later transitions to parenthood combined with strong ties to paid work were linked to reduced metabolic risk in mid-life. Fewer differences between work-family types and metabolic markers were seen for women.

## Introduction

When examining the importance of the interdependence of work and family life for health, studies have found that the combination of paid work with family responsibilities is associated with better health[1–6]. However this is not always the case[7,8]. Previous research in this area has largely focused upon women, for whom participation in paid work is particularly affected by caring for children[9,10]. The health benefits of employment have been well documented[11,12]. Also, married people tend to live longer, and report better health, than those who are not married[13–17], a difference that is starker for men. The timing of key life course events, such as the transition to parenthood, are likely to be important for health, with previous work showing that early parenthood is associated with increased cardiovascular risk[18] and mortality[19]. This may be particularly disadvantageous for health when combined with weak ties to paid work and partnerships[20]. Existing research into the importance of the combination of work and family life for health has been limited through the exclusion of men from analyses, the use of data on social roles from only one or two time points, and the reliance on subjective measures of health. Also the potential of early life factors, such as selection by health and socioeconomic factors, to influence work-family life courses and subsequent health has been little investigated.

Work-family life courses are thought to influence later health through biological and behavioural stress processes[21]. For instance, the multiple demands of combining paid work with parenting have been linked to poorer health in early studies[8]. However, more recent work has shown that weak ties to paid work and marriage, as well as early transitions to parenthood, are more often linked to increased physiological stress[11,14,20]. Part of this stress response might operate directly through altered physiological functioning (e.g. hypothalamo-pituitary-adrenal axis dysregulation and sympathetic-adrenal-medullary activation). More specifically, cortisol has also been shown to bind to glucocorticoid receptors on adipocytes in visceral fat, which can lead to triglyceride build up in visceral adipose tissue and hence increased central adiposity[22]. In turn visceral adiposity is pro-inflammatory, releasing cytokines such as interleukin-6 and tumour necrosis factor-α, resulting in changes in glucose and lipid metabolism and in the development of insulin resistance[23]. Mediation through differences in socioeconomic position[24], or an uptake, maintenance or increase in risky health behaviours[25–27], such as smoking, problem alcohol consumption and low physical activity levels, are also likely to play a role in explaining associations between work-family life courses and metabolic markers.

This paper focuses upon metabolic markers, which are known to be important indicators of insulin resistance, operating upstream from type II diabetes and cardiovascular disease[28–30]. We used a British birth cohort to characterise combined work and family life courses of both men and women using multichannel sequence analysis. These work-family life courses were then related to metabolic markers (waist circumference, blood pressure, high density lipoprotein (HDL) cholesterol, triglycerides, glycated haemoglobin (HbA_1c_)) in mid-life thought to mediate the association between stress and later health. The hypothesis was that strong ties to paid work and to partnership in combination with later transitions to parenthood would be associated with reduced metabolic risk (e.g. smaller waist circumference). The importance of early life circumstances in setting people onto more disadvantaged work-family life courses was also assessed. Finally, the potential mediating role of adult socioeconomic position and health behaviours were evaluated.

## Methods

### Data

This study used the UK Medical Research Council’s National Survey of Health and Development (NSHD), also known as the 1946 British birth cohort. The study received Multi-Centre Research Ethics Committee approval and written informed consent was provided by participants[31]. From an initial maternity survey of babies born during a single week in 1946, a stratified sample of 5,362 of all babies born to fathers in non-manual and agricultural employment and a quarter of births to fathers in manual employment was formed[32]. Our analyses were weighted to account for the stratified composition of the NSHD sample. Participants have been surveyed more than 23 times and information collected on economic, social, developmental and biological aspects[31]. At 53 years 3,035 (56.6%) participants were still part of the study (469 had died, 668 had permanently refused, 580 were living abroad, 330 were lost to follow-up and 280 temporarily refused the age 53 survey)[32].

### Measures

#### Metabolic markers

Data on waist circumference, blood pressure (systolic (SBP) and diastolic (DBP)), and blood samples for the assessment of triglycerides, HDL cholesterol, and HbA_1c_ were available at age 53. Blood samples were collected from non-fasted participants at age 53. Details of the biochemical procedures of triglyceride, HDL cholesterol and HbA_1c_ measurement have been published previously[33]. Participants were asked about medication status at time of blood sampling. Those taking medications affecting blood pressure (β-blockers, Calcium channel blockers, diuretics and drugs affecting the renin-angiotensin system) had their SBP values increased by 10mmHg and their DBP values increased by 5mmHg as recommended[34]. A similar procedure could not be followed for medications affecting HbA_1c_, triglycerides, and HDL cholesterol. Information on anti-diabetes and lipid-lowering medications was used to conduct sensitivity analyses in associations between work-family types and HbA_1c,_ triglycerides, and HDL cholesterol, respectively. The sensitivity analyses for HbA_1c_ involved running the analyses for all participants and then comparing these results to those when particpants taking anti-diabetic medications were removed. This was repeated for analyses involving triglycerides and HDL cholesterol using data on lipid-lowering medications.

#### Work-family life courses

Information on work, partnerships and parenthood was available at each adulthood survey (ages 19, 20, 22, 23, 25, 26, 31, 36, 43, and 53). Work, partnership and parenthood status variables were derived annually between ages 16 and 51 years (ending two years prior to metabolic risk evaluation at age 53). Work status was defined as full-time employment, part-time employment (≤30 hours/week), full-time homemaking, or other not employed (unemployed, sick, in education or other reason). Partnership status was defined as married, cohabiting, or not living with a partner. Parental status was categorised as no children in the household or youngest child >16 years, youngest child in the household <5 years, or youngest child in the household 5–16 years. These three life course domains were cross-classified to create 35 combined work-family state variables (one for each year between 16 and 51 years), each with 36 possible combinations of work, partnership and parenthood (4 work states x 3 partnership states x 3 parenthood states).

Sequence analysis was used to condense this detailed life course information into a work-family typology. This method measured the distance of each cohort member’s individual work-family sequence to a set of eight pre-defined model biographies. These model biographies were specified based upon previous knowledge of this cohort, and with a view to including as much variation across genders as possible whilst still maintaining adequate power (Table 1). Distances from each individual’s work-family sequence to the eight model biographies was calculated using the Dynamic Hamming approach[35], which is particularly appropriate when the timing of transitions is considered to be important. Participants were then categorised based upon their closest model biography, thereby creating a single work-family type variable with eight categories. Further information on how the work-family types were derived can be found in S1 Appendix and also in McMunn et al[36] and Lacey et al[20]. Estimates for work-family types containing fewer than 2% of men or women are not shown in subsequent results as these are unlikely to be reliable (Table 1).

**Table 1:** Distribution of Work-Family Life Course Types and Associated Model Biographies in the NSHD.

Work-family type	Men %^a^ (n = 1252)^b^	Women %^a^ (n = 1251)^b^	Model biography sequence
‘Work, early family’	47.7	15.1	Continuous full-time employment; married and children from early 20s
‘Work, marriage, non-parent’	7.9	9.0	Continuous full-time employment; married from early 20s; no children
‘Work, no family’	11.5	6.1	Continuous full-time employment; no partner or children
‘Work, later family’	30.6	3.5	Continuous full-time employment; cohabiting mid-20s, married from late 20s; children from early 30s
‘Later family, work break’	1.0^c^	11.6	Employed full-time until late 20s, homemaking from early 30s; married from mid 20s; children from early 30s
‘Early family, work break’	0.6^c^	14.6	Employed full-time until early 20s, homemaking from early-late 20s, employed part-time from early 30s; marriage and children from early 20s
‘Part-time work, early family’	0.7^c^	29.9	Employed full-time until early 20s, part time employed from early 20s; marriage and children from early 20s
‘No paid work, early family’	0.02^c^	10.3	Employed part-time until early 20s, homemaking from early 20s; marriage and children from early 20s

^a^ Results presented as percentages as are imputed data

^b^ Descriptives given for those with at least one observed outcome (n = 2,503)

^c^ Work-family groups containing fewer than 2% of participants are not presented in subsequent analyses as estimates are unlikely to be reliable

#### Covariates

Indicators of early life health and socioeconomic position (SEP) were included in this study to account for potential selections into different work-family types. At age 15 the cohort member’s parent was asked whether they had any concerns regarding the child’s health. Also information on internalising and externalising behaviours was derived from precursors of Rutter’s behavioural scales at ages 13 and 15, reported by the cohort member’s teacher. Factor analysis was used to derive a measure of internalising and externalising disorders by categorising scores based on established percentile cut-points[37]. For internalising scores: 0–50% (absent), 51–87% (mild) and ≥88% (severe). For externalising scores: 0–75% (absent), 76–93% (mild) and ≥94% (severe). Father’s social class (UK Registrar General’s Social Class schema) was used to indicate childhood SEP at age 4. Social class was categorised as professional and managerial (I), intermediate (II), skilled non-manual (IIINM), skilled manual (IIIM), semi-skilled (IV) or unskilled manual (V). Where this information was not available at age 4 information was taken from age 11 (n = 125) or age 15 (n = 48). Educational attainment was indicated by the highest qualification achieved by age 26 and categorised as no qualifications, secondary school education (Ordinary-level or Certificate of Secondary Education) or vocational training, advanced secondary education (Advanced-level or Burnham A2), degree or higher qualification.

Adult mediators included in this study were health behaviours, social class and body mass index (BMI) at age 53. Health behaviours considered were smoking status (never/ex/current), whether the cohort member participates in physical activity and problem drinking as indicated by the CAGE score (score of ≥2). The social class (Registrar General Social Class schema) of the head of household was used to indicate adult SEP. Height and weight were measured by the study nurses and BMI calculated as kg/m^2^.

### Statistical analysis

#### Missing data

Missing data are a particular problem for longitudinal studies, potentially resulting in bias, reduced sample sizes and loss of statistical power[38]. In this study missing information on work, partnerships and parenthood was imputed using a recommended method to overcome problems of collinearity and inaccurate estimation of missing sequence data[39]. Twenty imputed datasets were created. Multiple imputation by chained equations was then conducted to impute the covariates for those with complete work-family information following imputation (n = 2,513). The approach of imputation then deletion[40] was employed whereby all covariates were imputed for all cases before excluding those with missing data on each metabolic outcome. Descriptive analyses are presented for those with at least one observed outcome (n = 2,503).

#### Regression analyses

Ordinary least squares regression was used to test associations between work-family types and metabolic markers. Firstly the crude association was tested (model 1). Secondly we controlled for early life confounders–childhood SEP, educational attainment and child health (model 2). Finally we included potential adult mediators (health behaviours, adult SEP and BMI) in model 3. Models in which waist circumference was the outcome did not include BMI to avoid ‘over-adjusting’ for insulin resistance. As HbA_1c_, triglycerides and HDL cholesterol were log-transformed, results are presented as percentage difference to aid interpretation. All analyses were conducted using Stata version 13[41].

## Results

The distribution of work-family types in the NSHD is shown in Table 1. The majority (97.7%) of men were in work-family types characterised by continuous full-time employment; almost half (47.7%) of men combined this with early transitions to family life, whilst 30.6% made a later transition. Women’s work-family types were more diverse than men’s and women were more likely to occupy work-family types with weaker ties to paid work, for example long-term part-time employment (29.9%) and long-term full-time homemaking (10.3%). Further characteristics of the study sample are shown in S1 Table and S2 Table for men and women, respectively.

### Work-family types and metabolic markers in men

Table 2 shows the associations between work-family types and metabolic markers in men. Compared to men in the ‘Work, early family’ type, men who made later transitions to family life (‘Work, later family’) had smaller waist circumferences, which remained after controlling for early life factors (-2.16cm, 95% CI: -3.73, -0.59). The estimate did not change upon considering adult mediators of interest (adult SEP and heath behaviours). This was also the case for SBP for the same work-family type. The ‘Work, later family’ type also had lower DBP (-2.34mmHg, 95% CI: -4.15, -0.53) and triglycerides (9.78% lower, 95% CI: 0.81, 17.94), and in both cases this association was largely explained by the lower than average BMIs of this group. Men who were married but who were not parents (‘Work, marriage, non-parent’) had higher HDL cholesterol levels. This association was explained largely by differences in BMI. No differences in HbA_1c_ levels by work-family type were seen for men. The results for HDL cholesterol and triglycerides were robust to exclusion of those taking lipid-lowering medication (n = 43 and n = 37, respectively). Results for HbA_1c_ did not change upon removal of those taking anti-diabetes medications (n = 24).

**Table 2:** Associations Between Work-Family Life Courses and Metabolic Markers at Age 53 for Men.

	Model 1 –crude association		Model 2 –model 1 + early life factors		Model 3 –model 2 + adult mediators	
	**Regression coefficient**^**a**^	**95% CI**	**Regression coefficient**^**a**^	**95% CI**	**Regression coefficient**^**a**^	**95% CI**
***Waist circumference (n = 1244)***						
Work, early family	Ref		Ref		Ref	
Work, marriage, non-parent	-2.11	-4.51, 0.29	-1.56	-4.01, 0.89	-1.26	-3.73, 1.21
Work, no family	-1.56	-4.23, 1.10	-1.10	-3.74, 1.53	-0.40	-3.02, 2.23
Work, later family	-2.66	-4.23, -1.09	-2.16	-3.73, -0.59	-2.16	-3.71, -0.62
R-squared (%)	1.2		4.7		8.9	
***Systolic blood pressure (n = 1243)***						
Work, early family	Ref		Ref		Ref	
Work, marriage, non-parent	2.73	-2.48, 7.94	3.31	-1.90, 8.53	4.65	-0.46, 9.75
Work, no family	-3.51	-7.64, 0.62	-2.68	-6.84, 1.48	-1.09	-5.10, 2.91
Work, later family	-4.68	-7.55, -1.81	-4.03	-6.93, -1.13	-3.19	-6.00, -0.38
R-squared	1.8		5.6		12.7	
***Diastolic blood pressure (n = 1243)***						
Work, early family	Ref		Ref		Ref	
Work, marriage, non-parent	0.40	-2.57, 3.36	0.78	-2.23, 3.80	1.75	-1.04, 4.54
Work, no family	-1.93	-4.71, 0.86	-1.50	-4.25, 1.24	-0.34	-3.01, 2.32
Work, later family	-2.63	-4.44, -0.81	-2.34	-4.15, -0.53	-1.75	-3.52, 0.02
R-squared	1.5		5.3		12.9	
	**% difference**^**b**^	**95% CI**	**% difference**^**b**^	**95% CI**	**% difference**^**b**^	**95% CI**
***Triglycerides (n = 1033)***						
Work, early family	Ref		Ref		Ref	
Work, marriage, non-parent	-13.78	-25.72, 0.06	-12.37	-24.86, 2.19	-7.76	-19.85, 6.15
Work, no family	-12.65	-24.35, 0.88	-10.81	-22.42, 2.55	-2.91	-14.52, 10.28
Work, later family	-10.47	-18.32, -1.86	-9.78	-17.94, -0.81	-6.57	-14.67, 2.29
R-squared	1.4		4.5		16.2	
***HDL cholesterol (n = 1001)***						
Work, early family	Ref		Ref		Ref	
Work, marriage, non-parent	7.02	0.48, 13.99	7.23	0.68, 14.21	5.59	-1.02, 12.65
Work, no family	-5.09	-11.14, 1.37	-3.65	-10.89, 2.02	-5.86	-12.19, 0.93
Work, later family	3.54	-1.09, 8.39	3.06	-1.67, 8.03	3.07	-1.40, 7.74
R-squared	1.4		3.2		14.5	
***HbA1c (n = 1110)***						
Work, early family	Ref		Ref		Ref	
Work, marriage, non-parent	-0.73	-3.06, 1.65	0.02	-2.45, 2.55	0.56	-1.86, 3.05
Work, no family	2.01	-1.00, 5.07	2.28	-0.71, 5.37	2.77	-0.32, 5.95
Work, later family	-1.19	-2.79, 0.45	-0.55	-2.25, 1.16	-0.27	-1.94, 1.43
R-squared	0.9		4.3		5.9	

^a^Unstandardised regression coefficients

^b^ Results for triglycerides, HDL cholesterol and HbA_1c_ are presented as percentage difference as these outcomes were log-transformed

Model 1 –crude association

Model 2 additionally includes childhood social class, physical health, internalising and externalising disorders, and educational attainment

Model 3 additionally includes household social class, smoking status, physical activity, problem drinking, and BMI (except in regressions with waist circumference)

### Work-family types and metabolic markers in women

Associations between work-family life course types and metabolic markers for women are shown in Table 3. In general, fewer associations were seen for women than for men. Regarding waist circumference, women who were married but who didn’t have children (‘Work, marriage, non-parent’) had smaller waist circumferences (-4.67cm, 95% CI: -8.37, -0.97) than the reference group who had children (‘Work, early family’). This difference was not explained by the adult mediators of interest in this study. No other associations between work-family types and metabolic markers were seen for women. Results did not change during sensitivity analyses in which participants taking medications were removed (see results for men–same procedure followed, removing 15 and 12 women taking lipid-lowering medications in triglyceride and HDL cholesterol analyses, respectively. Also removing 21 women taking anti-diabetic medications in HbA_1c_ analyses).

**Table 3:** Associations Between Work-Family Life Courses and Metabolic Markers at Age 53 for Women.

	Model 1 –crude association		Model 2 –model 1 + early life factors		Model 3 –model 2 + adult mediators	
	**Regression coefficient**^**a**^	**95% CI**	**Regression coefficient**^**a**^	**95% CI**	**Regression coefficient**^**a**^	**95% CI**
***Waist circumference (n = 1244)***						
Work, early family	Ref		Ref		Ref	
Work, marriage, non-parent	-4.27	-8.02, -0.51	-4.67	-8.37, -0.97	-4.74	-8.50, -0.98
Work, no family	-1.90	-6.42, 2.63	-1.33	-5.82, 3.16	-0.46	-4.81, 3.90
Work, later family	-0.45	-6.20, 5.30	-0.35	-5.92, 5.21	0.28	-5.05, 5.60
Later family, work break	-0.47	-3.87, 2.93	-0.67	-3.98, 2.64	-0.31	-3.61, 3.00
Early family, work break	-0.89	-4.08, 2.30	-1.41	-4.54, 1.72	-1.26	-4.29, 1.77
Part-time work, early family	-1.76	-4.65, 1.12	-2.66	-5.52, 0.20	-2.14	-4.65, 0.66
No paid work, early family	-0.80	-4.99, 3.39	-1.73	-5.85, 2.40	-1.87	-5.95, 2.21
R-squared	0.9		5.2		10.1	
***Systolic blood pressure (n = 1213)***						
Work, early family	Ref		Ref		Ref	
Work, marriage, non-parent	2.43	-4.14, 8.99	1.54	-5.04, 8.12	3.07	-3.32, 9.47
Work, no family	-1.12	-7.91, 5.68	-1.08	-7.97, 5.81	-0.14	-7.11, 6.82
Work, later family	2.82	-6.27, 11.91	2.70	-6.65, 12.04	1.54	-7.41, 10.50
Later family, work break	-1.20	-6.35, 3.95	-1.62	-6.94, 3.69	-2.04	-7.49, 3.41
Early family, work break	2.90	-2.59, 8.38	2.26	-3.29, 7.81	2.39	-3.13, 7.91
Part-time work, early family	1.38	-2.89, 5.66	0.79	-3.62, 5.19	1.25	-3.21, 5.71
No paid work, early family	2.57	-3.24, 8.37	1.65	-4.21, 7.52	2.02	-3.67, 7.72
R-squared	0.2		1.3		8.1	
***Diastolic blood pressure (n = 1219)***						
Work, early family	Ref		Ref		Ref	
Work, marriage, non-parent	0.18	-3.51, 3.88	-0.24	-3.92, 3.45	0.77	-2.87, 4.41
Work, no family	0.29	-3.61, 4.18	0.09	-3.90, 4.07	0.76	-3.36, 4.88
Work, later family	0.56	-3.73, 4.84	0.32	-4.12, 4.77	-0.33	-4.68, 4.02
Later family, work break	-0.44	-3.28, 2.41	-0.66	-3.53, 2.21	-0.73	-3.64, 2.18
Early family, work break	-0.10	-3.07, 2.88	-0.26	-3.27, 2.74	-0.03	-3.05, 2.99
Part-time work, early family	0.10	-2.54, 2.74	-0.15	-2.83, 2.54	0.16	-2.49, 2.81
No paid work, early family	1.36	-2.14, 4.86	1.15	-2.42, 4.71	1.54	-1.95, 5.03
R-squared	0.2		1.2		8.4	
	**% difference**^**b**^	**95% CI**	**% difference**^**b**^	**95% CI**	**% difference**^**b**^	**95% CI**
***Triglycerides (n = 1448)***						
Work, early family	Ref		Ref		Ref	
Work, marriage, non-parent	13.43	-4.12, 34.21	13.99	-3.44, 34.57	24.09	6.68, 44.35
Work, no family	2.63	-14.09, 22.60	5.76	-11.44, 26.29	12.43	-4.24, 32.00
Work, later family	10.40	-14.86, 43.15	11.70	-14.18, 45.38	9.99	-13.12, 39.35
Later family, work break	-2.39	-14.12, 10.96	-1.78	-13.71, 11.79	1.19	-10.41, 14.30
Early family, work break	10.61	-3.34, 26.58	10.66	-3.37, 26.73	14.71	1.62, 29.47
Part-time work, early family	6.11	-5.68, 19.37	5.06	-6.66, 18.25	8.84	-2.18, 21.11
No paid work, early family	7.73	-9.72, 28.55	7.62	-9.52, 28.02	9.29	-5.91, 26.95
R-squared	0.7		2.6		19.3	
***HDL cholesterol (n = 1419)***						
Work, early family	Ref		Ref		Ref	
Work, marriage, non-parent	3.77	-4.65, 12.92	3.63	-4.71, 12.71	-0.14	-7.27, 7.53
Work, no family	2.40	-7.19, 12.99	1.13	-8.27, 11.50	-0.50	-9.22, 9.06
Work, later family	-6.63	-17.36, 5.50	-8.46	-19.54, 4.16	-8.86	-18.49, 1.91
Later family, work break	3.17	-4.35, 11.28	3.12	-4.56, 11.43	0.45	-6.78, 8.23
Early family, work break	0.60	-6.46, 8.19	0.48	-6.49, 7.97	-2.00	-8.29, 4.72
Part-time work, early family	-0.21	-6.63, 6.65	0.50	-5.87, 7.31	-1.07	-6.82, 5.02
No paid work, early family	0.16	-8.26, 9.36	0.87	-7.39, 9.86	-1.40	-8.57, 6.35
R-squared	0.8		4.7		19.6	
***HbA1c (n = 1500)***						
Work, early family	Ref		Ref		Ref	
Work, marriage, non-parent	-0.05	-3.58, 3.61	-0.24	-3.76, 3.41	0.75	-2.86, 4.50
Work, no family	1.73	-1.45, 5.01	1.89	-1.33, 5.21	2.48	-0.75, 5.82
Work, later family	-0.37	-3.14, 2.48	-0.61	-3.42, 2.28	-0.93	-3.82, 2.05
Later family, work break	-0.67	-3.07, 1.78	-0.77	-3.18, 1.71	-0.62	-3.10, 1.93
Early family, work break	-1.01	-3.28, 1.32	-1.07	-3.36, 1.28	-0.56	-2.74, 2.68
Part-time work, early family	0.80	-1.36, 3.01	0.51	-1.64, 2.72	0.83	-1.31, 3.03
No paid work, early family	3.09	-1.74, 8.16	2.69	-2.07, 7.67	2.76	-1.55, 7.27
R-squared	0.5		2.9		10.6	

^a^Unstandardised regression coefficients

^b^ Results for triglycerides, HDL cholesterol and HbA_1c_ are presented as percentage difference as these outcomes were log-transformed

Model 1—gender adjusted

Model 2 additionally includes gender, childhood social class, internalising and externalising disorders, and educational attainment

Model 3 additionally includes household social class, smoking status, physical activity, problem drinking, and BMI (except in regression with waist circumference)

## Discussion

Using a British birth cohort and an innovative method of characterising the work and family lives of British men and women, we found that later parenthood in combination with continuous full-time employment and marriage is associated with a more favourable metabolic risk profile (smaller waist circumference, lower blood pressure, and lower triglycerides) for men. The health advantage of later parenthood has previously been shown in this cohort[18] and in other studies[19,42,43]. However the present study adds to these findings by showing that it is later parenthood in combination with full-time employment which appears to be most beneficial for health. The link between early parenthood and higher blood pressure has been shown before in this cohort and was thought to be explained by increased stress resulting in prolonged sympathetic nervous system activation[18].

The more advantageous metabolic risk profiles of men entering parenthood later, at least in relation to SBP and waist circumference, were not fully explained by adult SEP, BMI or health behaviours in our study. It is therefore possible that there is a direct physiological response which does not operate through these factors. For instance, an increase in glucocorticoids, through hypothalamo-pituitary-adrenal axis dysregulation–one of the likely stress mechanisms linking social stressors, such as early family formation, to later health–may result in increased central adiposity through the differentiation and proliferation of adipocytes in visceral and abdominal adipose tissue. This has been shown in both animal and human studies[44–47].

It is possible that more accurately captured health behaviours measured over longer periods of time may play a role. Alternatively there may be a role of other mediators or residual confounding that we have not been able to consider in this study. For example, it is possible that other lifestyle factors are involved, such as diet, which we have not accounted for in this study but which are to some extent socially controlled within families[48].

Interestingly, women who were in this same work-family type (‘Work, later family’) did not have a better metabolic profile in mid-life than those who made an earlier transition to family life (‘Work, early family’–the reference group). This work-family group a smaller group of women (3.5% of women) and it is therefore possible that there was insufficient statistical power to detect a significant difference between this group and the reference. A power calculation was performed suggesting that 14.2% (approximately 177 women) would be required in this category to detect a statistical difference at the 80% power level. However the estimates appear to indicate little difference in metabolic markers. Also the findings for women are partly consistent with previous work by Hardy et al on the same cohort[18], which showed that women who had children earlier did not have significantly different DBP, waist:hip ratios, triglycerides, or HbA_1c_ levels than women who had children later. However they did find statistically significant differences in relation to SBP which we did not find in the present study. It is possible that any differences are diluted for women when additionally considering work and partnerships.

Men in the ‘Work, marriage, non-parent’ group had higher HDL cholesterol and women lower waist circumferences, indicative of lower metabolic risk. The association for HDL cholesterol amongst men appeared to be mediated by differences in BMI, however the smaller waist circumferences of women who did not have children was not fully explained by our mediators of interest. It is possible that there is a residual pregnancy effect; that women who do not have children retain a smaller waist circumference than their peers who do. Interestingly, in a previous study using the same cohort, we found that this work-family type had lower levels of life satisfaction at ages 60–64[6], suggesting a discrepancy between objective markers of health and subjective wellbeing.

The work-family life courses in this cohort were not found to be associated with differences in HbA_1c_ for men or women. This is consistent with previous work on this cohort,[18] in which no differences in HbA_1c_ were seen by age of parenthood.

### Methodological considerations

This study has a number of strengths and limitations. In the present study we were unable to take account of more detailed processes, such as work and relationship quality. It has been shown previously that these factors may be more important than role occupation[49,50]. Secondly, non-fasting blood samples were taken from participants. HDL cholesterol and HbA_1c_ do not require fasting to be accurate and reliable[51]; however, triglycerides are sensitive to fasting status. Despite this, non-fasting triglycerides have previously been highlighted as markers of insulin resistance and risk factors for myocardial infarction, ischaemic strokes and cardiovascular mortality[52].

This study has many strengths. For instance, this is one of the first studies to use multichannel sequence analysis to simultaneously consider the work, partnerships and parenthood histories of both men and women, recognising the interdependence of these important life course domains. The prospective longitudinal design enabled the inclusion of early life factors to account for potential selection into different work-family life courses. Results from this study are likely to be generalizable to British men and women of a similar age. Missing data were accounted for using multiple imputation, including an approach appropriate to categorical time series data. Finally, in contrast to many previous studies, work and family histories were linked to objective markers of health.

In conclusion, this study suggests that later parenthood combined with strong ties to paid work may result in a more favourable metabolic profile in mid-life for men. However, further research is needed into the detailed causal processes, such as qualitative aspects of work and family life (e.g. work stress and employment conditions), which might further explain these associations. Our findings also allude to the timing of parenthood as driving many of the associations seen here, and further research is needed to assess why this might be. Policies which promote the health of young parents and enable strong work ties may be fruitful in improving health.

## Supporting Information

S1 Appendix Further information on sequence analysis. (DOCX)

S1 Table Descriptive statistics of analysis variables by work-family type for NSHD men. (DOCX)

S2 Table Descriptive statistics of analysis variables by work-family type for NSHD women. (DOCX)
