# Supplementary material for: Work-Family Life Courses and Metabolic Markers in the MRC National Survey of Health and Development
Source: PLoS One. 2016 Aug 26;11(8):e0161923. doi: 10.1371/journal.pone.0161923 (PMC5001719; doi:10.1371/journal.pone.0161923)
Supplement: S1 Appendix — (DOCX) [file pone.0161923.s001.docx]

**Supplement 1.** Further information on sequence analysis

The three life course domains of work, partnerships and parenthood were considered simultaneously using multichannel sequence analysis,[1, 2] a relatively new method in epidemiology. Sequence analysis takes the whole work-family life course as the unit of analysis, allowing the analyst to condense a complex sequence of information into something more manageable for subsequent analyses (e.g. multivariable regression). Sequence analysis compares each individual’s sequence to that of every other participant in the dataset and results in a list of distance measures. These distances represent the similarity or every individual’s life course sequence. In this study, Lesnard’s Dynamic Hamming algorithm[3] was used to calculate distances. This method is particularly appropriate to the current study, where the timing of life course transitions is thought to be important. In particularly it assigns higher costs to substitutions that parallel transitions made at time when relatively few participants are changing states. Conversely, lower costs are assigned to substitutions paralleling transitions made at times when many participants are changing states (e.g. in later adolescence). Dynamic Hamming sequence analysis was implemented using the –seqcomp- plug-in in Stata.[4]

The eight model biographies detailed in table 1 were constructed by the first and last authors independently and then compared. The overlap between derived model biographies was found to be substantial. These model biographies were then added to the dataset as ‘invented’ cases and every participant’s work-family life courses were compared to that of each model biography. Participants were allocated to the model biography group which was closest to their actual work-family life course type, based on the distance list derived, thereby creating a single work-family type variable with eight categories. This was then used in subsequent analyses presented in this study. The validity of this classification was assessed by examining between- and within-group variability.

**References**

1. Gauthier J-A, Widmer E, Bucher P, Notredame C (2010) Multichannel Sequence Analysis Applied To Social Science Data. Sociol Methodol 40:1–38.

2. Pollock G (2007) Holistic trajectories: a study of combined employment, housing and family careers by using multiple-sequence analysis. J R Stat Soc Ser A (Statistics Soc 170:167–183.

3. Lesnard L (2010) Setting Cost in Optimal Matching to Uncover Contemporaneous Socio-Temporal Patterns. Sociol Methods Res 38:389–419.

4. Lesnard L (2008) seqcomp, a sequence analysis Stata plug-in.
