# Supplementary material for: Work-Family Life Courses and Metabolic Markers in the MRC National Survey of Health and Development
Source: PLoS One. 2016 Aug 26;11(8):e0161923. doi: 10.1371/journal.pone.0161923 (PMC5001719; doi:10.1371/journal.pone.0161923)
Supplement: S1 Table — (DOCX) [file pone.0161923.s002.docx]

**Supplement 1 Table.** Descriptive statistics of analysis variables by work-family type for NSHD men (n=1,252)

|  | **Work, early family** | **Work, marriage, non-parent** | **Work, no family** | **Work, later family** |
| --- | --- | --- | --- | --- |
| Waist circumference (cm) - mean (SD) | 99.2 (10.4) | 97.0 (10.1) | 97.4 (12.3) | 96.5 (10.3) |
| Triglycerides (mmol/L) - median [IQR] | 2.0 [1.5, 3.2] | 1.8 [1.3, 2.7] | 1.9 [1.2, 3.0] | 1.9 [1.4, 2.6] |
| HDL cholesterol (mmol/L) - median [IQR] | 1.4 [1.1, 1.6] | 1.5 [1.2, 1.8] | 1.3 [1.1, 1.6] | 1.4 [1.2, 1.7] |
| Systolic BP (mmHg) - mean (SD) | 143.4 (19.8) | 146.1 (21.6) | 139.9 (19.8) | 138.7 (18.4) |
| Diastolic BP (mmHg) - mean (SD) | 89.3 (12.1) | 89.5 (12.1) | 87.4 (13.2) | 86.7 (11.9) |
| HbA_1c_ (%) - median [IQR]  HbA_1c_ (mmol/mol) | 5.6 [5.4, 5.9]  37.7 [35.5, 41.0] | 5.5 [5.3, 5.8]  36.6 [34.4, 39.9] | 5.6 [5.4, 5.9]  37.7 [35.5, 41.0] | 5.6 [5.3, 5.8]  37.7 [34.4, 39.9] |
| ***Early life factors*** |  |  |  |  |
| Physical health problem, % |  |  |  |  |
| No | 91.1 | 88.5 | 86.0 | 95.1 |
| Yes | 8.9 | 11.5 | 14.0 | 4.9 |
| Internalising disorders, % |  |  |  |  |
| Absent | 62.4 | 60.5 | 45.2 | 53.6 |
| Mild | 30.3 | 30.6 | 40.2 | 36.4 |
| Severe | 7.3 | 8.9 | 14.6 | 10.0 |
| Externalising disorders, % |  |  |  |  |
| Absent | 69.7 | 79.1 | 75.7 | 73.9 |
| Mild | 21.1 | 17.6 | 17.4 | 18.7 |
| Severe | 9.3 | 3.4 | 6.9 | 7.4 |
| Childhood social class (RGSC) |  |  |  |  |
| I | 1.6 | 7.1 | 7.4 | 5.1 |
| II | 16.1 | 10.9 | 13.6 | 16.7 |
| IIINM | 8.4 | 10.1 | 7.2 | 11.8 |
| IIIM | 46.5 | 35.9 | 38.9 | 40.2 |
| IV | 19.5 | 27.1 | 19.3 | 16.0 |
| V | 7.9 | 9.0 | 13.6 | 10.2 |
| Educational attainment |  |  |  |  |
| No qualifications | 44.3 | 33.2 | 49.7 | 32.6 |
| CSE/O-level | 24.5 | 17.5 | 18.9 | 20.8 |
| A-level | 23.2 | 34.0 | 19.6 | 32.0 |
| Higher qualification/degree | 8.0 | 15.4 | 11.8 | 14.6 |
| ***Adult mediators (53 yrs)*** |  |  |  |  |
| Household social class (RGSC) |  |  |  |  |
| I | 7.5 | 14.7 | 6.6 | 16.9 |
| II | 36.6 | 34.0 | 26.2 | 39.1 |
| IIINM | 8.3 | 14.7 | 13.8 | 9.3 |
| IIIM | 35.1 | 27.4 | 32.9 | 29.4 |
| IV | 9.6 | 4.2 | 15.7 | 4.5 |
| V | 2.9 | 5.0 | 4.8 | 0.8 |
| Smoking status |  |  |  |  |
| Never smoked | 27.2 | 42.7 | 45.7 | 38.1 |
| Ex-smoker | 46.9 | 27.0 | 24.6 | 40.1 |
| Current smoker | 25.9 | 20.3 | 29.7 | 21.9 |
| Problem drinking |  |  |  |  |
| CAGE score <2 | 88.3 | 95.1 | 94.4 | 92.0 |
| CAGE score ≥2 | 11.7 | 4.9 | 5.6 | 8.0 |
| Exercises regularly |  |  |  |  |
| Yes | 49.9 | 39.2 | 60.7 | 46.6 |
| No | 50.1 | 60.8 | 39.4 | 53.4 |
| BMI (kg/m^2^) - mean (SD) | 28.1 (3.9) | 27.1 (3.6) | 26.7 (4.2) | 27.2 (3.8) |

SD = standard deviation; IQR – interquartile range
