# Supplementary material for: Work-Family Life Courses and Metabolic Markers in the MRC National Survey of Health and Development
Source: PLoS One. 2016 Aug 26;11(8):e0161923. doi: 10.1371/journal.pone.0161923 (PMC5001719; doi:10.1371/journal.pone.0161923)
Supplement: S2 Table — (DOCX) [file pone.0161923.s003.docx]

**Supplement 2 Table.** Descriptive statistics of analysis variables by work-family type for NSHD women (n=1,251)

|  | **Work, early family** | **Work, marriage, non-parent** | **Work, no family** | **Work, later family** | **Later family, work break** | **Early family, work break** | **Part-time work, early family** | **No paid work, early family** |
| --- | --- | --- | --- | --- | --- | --- | --- | --- |
| Waist circumference (cm) - mean (SD) | 87.8 (13.1) | 83.6 (12.7) | 85.9 (14.6) | 87.4 (12.9) | 87.3 (12.6) | 86.9 (12.2) | 86.0 (12.7) | 87.0 (15.5) |
| Triglycerides (mmol/L) - median [IQR] | 1.4 [1.1, 2.0] | 1.5 [1.2, 2.3] | 1.4 [1.0, 2.3] | 1.4 [1.0, 2.3] | 1.4 [1.1, 2.0] | 1.6 [1.2, 2.2] | 1.4 [1.1, 2.1] | 1.5 [1.1, 1.9] |
| HDL cholesterol (mmol/L) - median [IQR] | 1.8 [1.4, 2.1] | 1.8 [1.6, 2.1] | 1.8 [1.5, 2.1] | 1.6 [1.4, 2.0] | 1.9 [1.5, 2.1] | 1.8 [1.5, 2.1] | 1.8 [1.4, 2.1] | 1.8 [1.5, 2.1] |
| Systolic BP (mmHg) - mean (SD) | 133.6 (20.4) | 136.4 (22.6) | 132.5 (20.3) | 136.5 (23.5) | 132.9 (19.7) | 136.9 (23.5) | 135.3 (18.8) | 137.0 (20.7) |
| Diastolic BP (mmHg) - mean (SD) | 82.6 (11.1) | 82.9 (12.6) | 82.9 (12.3) | 83.3 (10.9) | 82.3 (11.0) | 82.7 (12.5) | 82.8 (12.3) | 84.3 (12.9) |
| HbA_1c_ (%) - median [IQR]  HbA_1c_ (mmol/mol) | 5.6 [5.4, 5.8]  37.7 [35.5, 39.9] | 5.6 [5.4, 5.8]  37.7 [35.5, 39.9] | 5.6 [5.4, 5.8]  37.7 [35.5, 39.9] | 5.5 [5.4, 5.7]  36.6 [35.5, 38.8] | 5.5 [5.3, 5.8]  36.6 [34.4, 39.9] | 5.5 [5.3, 5.8]  36.6 [34.4, 39.9] | 5.6 [5.4, 5.9]  37.7 [35.5, 41.0] | 5.6 [5.4, 5.9]  37.7 [35.5, 41.0] |
| ***Early life factors*** |  |  |  |  |  |  |  |  |
| Physical health problem, % |  |  |  |  |  |  |  |  |
| No | 91.8 | 95.4 | 88.9 | 96.2 | 91.4 | 89.6 | 92.5 | 89.8 |
| Yes | 8.2 | 4.6 | 11.1 | 3.9 | 8.6 | 10.4 | 7.5 | 10.2 |
| Internalising disorders, % |  |  |  |  |  |  |  |  |
| Absent | 61.0 | 54.7 | 38.0 | 52.7 | 35.5 | 51.7 | 43.7 | 37.0 |
| Mild | 29.2 | 30.4 | 45.0 | 35.3 | 43.0 | 39.5 | 42.6 | 43.0 |
| Severe | 9.8 | 14.8 | 17.0 | 12.0 | 21.5 | 8.9 | 13.7 | 20.0 |
| Externalising disorders, % |  |  |  |  |  |  |  |  |
| Absent | 76.2 | 81.5 | 78.0 | 82.0 | 80.8 | 83.1 | 79.4 | 70.7 |
| Mild | 13.7 | 14.8 | 13.6 | 16.4 | 17.4 | 12.4 | 14.5 | 24.3 |
| Severe | 10.2 | 3.7 | 8.4 | 1.6 | 1.8 | 4.5 | 6.1 | 5.1 |
| Childhood social class (RGSC) |  |  |  |  |  |  |  |  |
| I | 1.4 | 3.9 | 7.2 | 3.9 | 5.2 | 1.4 | 2.8 | 2.2 |
| II | 10.7 | 13.6 | 23.5 | 29.4 | 14.5 | 12.2 | 14.4 | 14.5 |
| IIINM | 13.5 | 3.6 | 15.9 | 9.1 | 10.0 | 13.2 | 7.3 | 10.4 |
| IIIM | 44.1 | 58.2 | 27.5 | 38.9 | 43.4 | 40.5 | 46.8 | 46.2 |
| IV | 25.4 | 12.6 | 17.9 | 16.5 | 23.8 | 24.4 | 18.7 | 15.8 |
| V | 4.9 | 8.1 | 8.1 | 2.2 | 3.1 | 8.3 | 10.1 | 10.9 |
| Educational attainment |  |  |  |  |  |  |  |  |
| No qualifications | 37.0 | 40.1 | 29.2 | 36.6 | 29.1 | 43.9 | 53.4 | 54.0 |
| CSE/O-level | 36.8 | 42.7 | 38.8 | 32.8 | 40.5 | 40.6 | 30.4 | 34.2 |
| A-level | 24.1 | 14.5 | 23.3 | 27.5 | 26.6 | 13.0 | 13.5 | 9.2 |
| Higher qualification/degree | 2.2 | 2.8 | 8.8 | 3.1 | 3.7 | 2.6 | 2.7 | 2.6 |
| ***Adult mediators*** |  |  |  |  |  |  |  |  |
| Household social class (RGSC) |  |  |  |  |  |  |  |  |
| I | 4.2 | 3.9 | 2.6 | 12.1 | 15.3 | 4.7 | 8.9 | 9.7 |
| II | 35.1 | 36.3 | 37.0 | 42.8 | 42.4 | 38.9 | 30.4 | 35.9 |
| IIINM | 12.1 | 18.4 | 26.1 | 16.3 | 14.7 | 11.3 | 10.5 | 10.0 |
| IIIM | 23.7 | 21.1 | 21.1 | 23.0 | 21.1 | 29.8 | 31.4 | 23.3 |
| IV | 14.6 | 17.8 | 7.5 | 5.4 | 3.5 | 9.3 | 13.5 | 14.8 |
| V | 10.3 | 2.6 | 5.6 | 0.4 | 3.0 | 5.9 | 5.4 | 6.4 |
| Smoking status |  |  |  |  |  |  |  |  |
| Never smoked | 41.5 | 48.7 | 51.5 | 42.9 | 60.3 | 48.5 | 45.3 | 53.8 |
| Ex-smoker | 24.9 | 25.8 | 23.4 | 37.9 | 30.7 | 30.5 | 27.8 | 23.1 |
| Current smoker | 33.6 | 25.6 | 25.0 | 19.3 | 9.0 | 21.1 | 26.9 | 23.1 |
| Problem drinking |  |  |  |  |  |  |  |  |
| CAGE score <2 | 93.1 | 96.1 | 98.3 | 96.8 | 93.4 | 95.4 | 95.7 | 96.4 |
| CAGE score ≥2 | 6.9 | 3.9 | 1.7 | 3.3 | 6.6 | 4.7 | 4.3 | 3.7 |
| Exercises regularly |  |  |  |  |  |  |  |  |
| Yes | 55.7 | 61.9 | 46.4 | 44.2 | 44.3 | 55.4 | 52.2 | 60.9 |
| No | 44.3 | 38.1 | 53.6 | 55.8 | 55.7 | 44.6 | 47.8 | 39.1 |
| BMI (kg/m^2^) - mean (SD) | 27.9 (5.1) | 26.7 (5.3) | 27.6 (5.9) | 28.9 (7.4) | 28.3 (5.5) | 27.9 (4.9) | 27.7 (5.4) | 28.3 (6.4) |

SD = standard deviation; IQR – interquartile range
